# Traditional Uses, Phytochemistry, and Bioactivities of *Mesosphaerum suaveolens* (L.) Kuntze

**DOI:** 10.1155/2022/3829180

**Published:** 2022-03-10

**Authors:** José Weverton Almeida-Bezerra, Felicidade Caroline Rodrigues, José Jailson Lima Bezerra, Anderson Angel Vieira Pinheiro, Saulo Almeida de Menezes, Aline Belém Tavares, Adrielle Rodrigues Costa, Priscilla Augusta de Sousa Fernandes, Viviane Bezerra da Silva, José Galberto Martins da Costa, Rafael Pereira da Cruz, Maria Flaviana Bezerra Morais-Braga, Henrique Douglas Melo Coutinho, Edward Teixeira de Albergaria, Marcos Vinicius Meiado, Abolghasem Siyadatpanah, Bonglee Kim, Antônio Fernando Morais de Oliveira

**Affiliations:** ^1^Federal University of Pernambuco–UFPE, Recife 50670-901, PE, Brazil; ^2^Federal University of Paraíba–UFPB, João Pessoa 58051-970, PB, Brazil; ^3^Regional University of Cariri–URCA, Crato 63105-000, CE, Brazil; ^4^Federal University of Sergipe–UFS, Itabaiana 49500-000, SE, Brazil; ^5^Ferdows School of Paramedical and Health, Birjand University of Medical Sciences, Birjand, Iran; ^6^Department of Pathology, College of Korean Medicine, Kyung Hee University, Seoul 02447, Republic of Korea

## Abstract

*Mesosphaerum suaveolens* (L.) Kuntze is a species widely used traditionally in the treatment of ailments, such as stomach pain, hemorrhoids, cough, verminosis, ulcer, liver disease, fever, influenza, nasal congestion, and inflammation. This review aims to provide a survey of available information on seven international electronic databases (Google Scholar, Medline, ResearchGate, Web of Science, Scopus, Science Direct, and PubMed) about botanical aspects, traditional uses, phytochemistry, and biological activities of *M. suaveolens*. *Mesosphaerum suaveolens* is a tropical America native species, but it can be found in several parts of the world as a ruderal plant. The species is the most studied species of the genus Lamiaceae due its phytochemical aspect, especially regarding the chemical composition of its essential oil. Besides the essential oils, *M. suaveolens* is a source of numerous secondary compounds such as triterpenes, diterpenes, and phenolic compounds, which are related to its biological activities, such as allelopathic, antibacterial, antifungal, insecticidal, and larvicidal activities as described in the literature.

## 1. Introduction

Plant species, with medicinal properties that have always been part of human life, are being used both for the treatment of diseases as for food. For the treatment of diseases, they are accessible and culturally accepted, so their use is popular since ancient civilizations [1, 2]. The Lamiaceae family is one of the most diverse and widespread in terms of the ethnomedicinal value and variety of plants with biological and medical applications [3–8]. Regarding the genus *Mesosphaerum*, previous studies demonstrate the ethnomedicinal and pharmacological importance of some species that belong to it, such as *Mesosphaerum sidifolium* (L'Hérit.) Harley & J.F.B. Pastore, used to treat stomach disorders and headaches, as well as being used as an expectorant, carminative, and tonic. This species possesses in vivo antitumor activity against Ehrlich ascites carcinoma cells causing growth inhibition by inducing cell cycle arrest, besides not showing cytotoxicity [9]. Another species with several bioactivities is *Mesosphaerum verticillatum* (syn. *Hyptis verticillata* Jacq.) with anti-inflammatory, antimicrobial, and anticancer potentials, among other reports. Ethnomedicinal uses of this plant include cough, colds, asthma, fever, tonsillitis, uterine fibroids, bronchitis, and gastrointestinal problems [5].

In Northeast Brazil, the use of plant species as therapeutic resources is widespread, and one species present in this region is *Mesosphaerum suaveolens* (L.) Kuntze, known as “bamburral,” “erva-canudo,” or “alfazema-brava” [10]. Its leaves are mainly used to treat respiratory diseases (asthma, bronchitis, colds, and flu) and diseases related to the gastrointestinal tract [1]. Such medicinal uses are related to the chemical heterogeneity arising from the secondary metabolism of the species, a recurrent characteristic in species of the Lamiaceae family [6].

Several works in the literature indicate that *M. suaveolens* presents a high biotechnological potential, mainly regarding its essential oil [10]. In addition, a large number of studies have emphasized the biological activities of the essential oil and extracts of this species against pathogenic microorganisms to humans [6]. Taking into account that *M. suaveolens* is a medicinal species widely studied by the scientific community, the main objective of this work was to make a general review of the botanical aspects, traditional uses, phytochemistry, toxicity and biological, and pharmacological activities.

## 2. Materials and Methods

Methodologically, it was used the keywords “*Mesosphaerum suaveolens*” and its synonym “*Hyptis suaveolens*” associated to the terms “biological activity,” “bioactive,” “ethnomedicinal use,” “traditional use,” “ethnobotany,” “ethnopharmacology,” “toxicity,” “natural products,” “phytochemistry,” and “allelopathy” to collect information available on Google Scholar, Medline, ResearchGate, Web of Science, Scopus, ScienceDirect, and PubMed databases. The consideration insertion criteria of the articles were as follows: full article only, articles written in English and/or Portuguese languages, and all available and opened access articles, with no time limit determined.

It was obtained 190 articles dated between 1971 and 2021 which were grouped into some categories. (1) Botanical aspects, with information on description, classification, and geographical distribution; (2) phytochemistry; (3) ethnobotany; (4) biological activities; and (5) pharmacological activities. The trial process (collecting of the articles, reading of the abstracts, and checking the insertion criteria) took three months, and all the selected articles had been read completely and summarized in a table with the isolated chemical constituents and their respective biological activities.

## 3. Review

### 3.1. Botanical Aspects: Description, Classification, and Distribution

*Mesosphaerum suaveolens* (L.) Kuntze is an herbaceous plant belonging to the Lamiaceae family. The word “*mesosphairon*” comes from the Greek and Latin “*mesosphaerum*,” meaning “a type of tuberose with medium-sized leaves,” while its specific epithet *suaveolens*, means “with a sweet fragrance” due to the aroma of essential oils exhaled by the trichomes present on its leaves [11, 12].

Its vernacular name varies widely according to the region of occurrence. In the northeastern region of Brazil, the species is known as “bamburral” and “alfazema-brava” and in the southern region of the country, the herbaceous plant is called “erva-canudo” and “betônica-brava” [10]. In other parts of the world, such as in India, it is known as “pignut,” “beejabandha,” “sima tulasi,” “sakavong,” “pichi tulas,” and “bushmint” [13], while in Nigeria, it is known as “false buttonweed” and in Bangladesh as “tukma” [14]. In French-speaking countries, the species is called “horehound,” “pignut,” “wild spikenard,” “gros baume,” and “Hyptis à odeur.” In other languages, the plant is called “chao,” “hierba de las muelas,” “menta de campo” (Spanish), “wilaiti tulsi” (Hindi), “bhustrena,” “darp tulas,” “jungli tulas” (Marathi), “sirna tulasi” (Telugu), “bilati tulas” (Bengali), “ganga tulasi” (Ora), and “bhustrena” (Sanskrit) [13].

Taxonomically, *M. suaveolens* presents as botanical synonyms *Ballota suaveolens* L., *Hyptis suaveolens* (L.) Poit., *Bystropogon suaveolens* (L.) L'Hér., *Bystropogon graveolens* Blume, *Hyptis congesta* Leonard, *Hyptis ebracteata* R.Br., *Hyptis plumierii* Poit., and *Marrubium indicum* Blanco, with *H. suaveolens* as the most widespread synonym in scientific circles. However, the current circumscription of the genus *Mesosphaerum* P. Browne was recognized in 2012 after phylogenetic studies [15].

**Figure 1:**
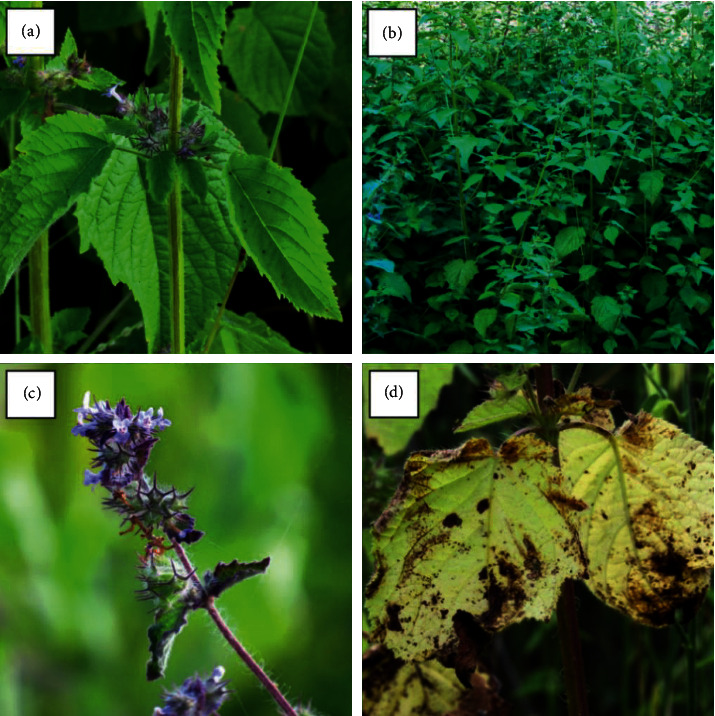
*Mesosphaerum suaveolens* (L.) Kuntze (Lamiaceae). (a) Leaves and stem. (b) Population of *M. suaveolens* in an area of Caatinga, a seasonally dry tropical forest, in Quixelô-CE, Brazil. (c) Prominence of flowers. (d) Leaves in senescence. Source: author (2018).

Morphologically, *M. suaveolens* is an erect herb or subshrub that measures up to 2 m in height. Its photosynthetic quadrangular stem is hairy with closely spaced branches and nodes. It has oval leaves, serrate or cordate margin, pilose limb, acute apex, and obtuse base with opposite crossed phyllotaxis. The petioles are short, canaliculate, as are its stems. Its inflorescences consist of up to 20 flowers located around the nodes and near the leaf axils. The flowers are pedunculate, with a persistent, tubular calyx, and 5 pointed sepals. The corolla is also tubular with five lilac petals, and the lobes are evident. Its fruits are dry, indehiscent, and uniseminated, originating from a bicarpellate gynoecium. Such fruits originate dimorphic seeds, two per fruit. Morphologically, such diaspores are elongated with dorsoventral flattening, longitudinal median ridge, starting near the hilum and extending to the apex of the seed with retusa boundary with black coloration (Figure 1) [16–18].

As for the geographical distribution, *M. suaveolens* is native to tropical America; however, as it is ruderal, it ended up invading natural ecosystems in tropical and subtropical regions of the globe, so that, due to this widespread occurrence, the species is considered a pantropical ruderal species [19–22]. In Brazil, *M. suaveolens* is present in almost the entire territory [23].

### 3.2. Phytochemistry

*Mesosphaerum suaveolens* is an important source of essential oils, alkaloids, flavonoids, phenols, saponins, triterpenes, and sterols [13, 24]. The essential oil of this species, obtained exclusively from its leaves, has already been chemically characterized in several studies. However, since this species exhibits a high level of genetic polymorphism and allows adaptation to changes in environmental characteristics, high variability in the composition and content of the major constituents (>20%) has been found [25]. In *M. suaveolens* extracts, terpenoids had a great predominance (mono, di, tri, and sesquiterpenes) (Table 1) (Table 1). Among the diterpenes, suaveolic acid stood out with recognized antimicrobial and allelopathic action [26]. Furthermore, phenolic acids, phenylpropanoids, flavonoids [10, 27], and fatty acids [28, 29] were also identified in different parts of *M. suaveolens* (Table 1).

### 3.3. Ethnobotany

Traditionally, *M. suaveolens* is taken to treat ailments in Brazil, Benin, India, Nigeria, Thailand, and Togo. In Brazil, the leaves in the form of infusions, decoctions, teas, and syrups are used to treat ulcers, inflammation, respiratory diseases (asthma, bronchitis, colds, flu, and sinusitis), diseases related to the gastrointestinal tract, pain, dizziness, nausea, nervousness, and constipation [1, 56–64]. The leaves are also used to treat headaches [65], malaria [14, 66], fever [67, 68], and used to reduce labor time and labor pain [69]. The flowers of *M. suaveolens* are employed as therapeutic resources against dysmenorrhea, respiratory diseases, and as a febrifuge [70, 71].

In the Asian continent, more specifically in India, the leaves, stems, inflorescence, and roots are used to treat urinary calculi [72], stomach pain [73], healing, itching [74], boil, eczema, diabetes [75], pneumonia [76], and fever [77]. Besides that, the seeds of *M. suaveolens* are used to treat gynecological disorders such as menorrhagia, leucorrhea, and rheumatism [78, 79]. The fresh poultice of the leaves is applied to snake bites, wounds, and mycoses [80], while the paste of the fresh leaves is also indicated for skin diseases [81].

In South Asia, in Bangladesh, traditional communities use the seeds in juice preparations to treat constipation and weakness [82, 83]; in addition, the seeds are consumed along with roots of *Bombax ceiba* to treat gonorrhea [84, 85], and the paste of the leaves is used to treat skin infections [86]. In Togo, the leaves of the species are spent in decoction form for the treatment of gynecological disorders [87], while in Thailand, the decoction of the roots is indicated in cases of food poisoning [88]. On the African continent, more specifically in Benin and Nigeria, the whole plant of *M. suaveolens* is used for the treatment of candidiasis and as a blood tonic [89, 90].

From a veterinary point of view, *M. suaveolens* has also been used for the treatment of diseases in animals. Such use is reported in India for the treatment of inflammation in cattle, with the juice of the leaves being applied to the animal's eyes [91]. In Brazil, the species is employed against diarrhea [92]. In the African continent, more specifically in Kenya, the aerial parts of the plant are utilized as a repellent for the mosquito *Anopheles gambiae* Giles, 1926 (Diptera: Culicidae) [93, 94].

### 3.4. Biological Activities

#### 3.4.1. Allelopathic Activity

According to Sharma et al. [95], after the establishment of *M. suaveolens* in an area, it becomes evident that the species imposes a profound impact on the local vegetation, as the number of species, richness, diversity, and uniformity is severely reduced. Although *M. suaveolens* is native to the Brazilian territory, it is distributed in different ecosystems, such as Caatinga, a seasonally dry tropical forest [20].

*Mesosphaerum suaveolens* produces numerous seeds of rapid germination and subsequent growth and thus manages to occupy and dominate environments because of its allelopathic action [96]. Islam et al. [26], for example, isolated suaveolic acid from *M. suaveolens* and demonstrated in bioassays that this diterpene exhibits allelopathic action, interfering with the growth of the caulicle and radicle of *Lepidium sativum* L. (Brassicaceae), *Lactuca sativa* L. (Asteraceae), *Lolium multiflorum* Lam. (Poaceae), and *Echinochloa crus-galli* (L.). P. Beauv. (Poaceae). Their extracts present allelopathic action against *Echinochloa crus-galli* (L.) P. Beauv. [97], *Sorghum vulgare* Pers., *Raphanus sativus* L., and *Lactuca sativa* L. [98].

Allelochemicals present in the species have been reported to act by causing oxidative stress, reduction in chlorophyll content, and inducing the formation of chromosomal aberrations [99, 100]. Such damage may occur in response to the synergistic action of the constituents.

In addition to heterotoxicity, *M. suaveolens* has been found to exhibit autotoxicity; however, its constituents affect other species more than itself [101]. Thus, the low amounts of allelochemicals released by *M. suaveolens* affect the ecological succession of other species, but do not affect the species itself as much.

Despite reports of the allelopathic action of *M. suaveolens*, it is worth noting that most of these studies were conducted under laboratory conditions and with extracts of the plant, so these actions do not match the allelopathic actions found in the environment. Thus, it is necessary to conduct studies that simulate as much as possible the natural conditions, to affirm whether one species can affect another. Only Kapoor [99] evaluated the allelopathic action of *M. suaveolens* in conditions similar to those found in the environment, demonstrating in fact that the species has allelopathic action on *Parthenium hysterophorus* L. (Asteraceae).

*M. suaveolens* has allelochemicals from its secondary metabolism, which compromise the structure and plant diversity [102].

#### 3.4.2. Antimicrobial Activity

Teas of *M. suaveolens* are used to treat diseases related to the gastrointestinal and respiratory tracts [1], so numerous researchers have hypothesized that the species exhibits biological activity against strains of pathogenic microorganisms.

Cyrille et al. [103] evaluated the antibacterial action of the hydroethanolic extract (70%) of the leaves and found that the species presented low antibacterial activity since an MIC of 3.12 mg/ml was observed against *Staphylococcus aureus* (Rosenbach, 1884) (Staphylococaceae) ATCC 25923 and *Pseudomonas aeruginosa* (Schroeter, 1872) (Pseudomonadaceae) ATCC 27853 strains. It is worth noting that MIC values obtained above 1 mg/ml (1000 *μ*g/ml) do not reflect clinically notable activity (Van Vuuren, 2008) (Table 2).

The works evaluating the antimicrobial action of the species highlight the use of the volatile terpenes (essential oils) of the leaves (Table 2). Xu et al. [104] demonstrated that the oil of *M. suaveolens* showed antimicrobial action through the microdilution technique with MIC values of great clinical relevance, notably against *Bacillus subtilis* (Cohn, 1872) (Bacillaceae) CMCC 63501, *Escherichia coli* (T. Escherich, 1885) (Enterobacteriaceae) CMCC 44102, and *Botrytis cinerea* (De Bary) Whetzel, 1945 (Sclerotiniaceae). The oil was also very active against *S. aureus* CMCC 26001, *P. aeruginosa* CMCC 10104, *Fusarium graminearum* Schwabe, 1839 (Nectriaceae), *Exerohilum turcicum* (Pass.) K.J. Leonard & Suggs, 1974 (Pleosporaceae), and *Lecanosticta acicola* (Thüm.) Syd., 1924 (Mycosphaerellaceae).

Besides the essential oil, the fixed oil from *M. suaveolens* seeds showed activities of clinical relevance against *E. coli* MTCC 443 (MIC 0.5 mg/ml), *Salmonella typhi* Typhi (Enterobacteriaceae) MTCC 531 (MIC 0.125 mg/ml), *Shigella flexneri* Castellani & Chalmers, 1919 (Enterobacteriaceae) MTCC 1457 (MIC 0.5 mg/ml), *Vibrio vulnificus* (Reichelt et al.) Farmer, 1980 (Vibrionaceae) MTCC 1145 (MIC 0.5 mg/ml), *P. aeruginosa* MTCC 424 (MIC 0.125 mg/ml), *Lactobacillus plantarum* (Orla-Jensen) Bergey et al., 1923 (Lactobacillaceae) MTCC 2621 (MIC 0.125 mg/ml), *Lactobacillus leishmanii* (Henneberg) Bergey et al. 1923 (Lactobacillaceae) MTCC 911 (MIC 0.25 mg/mL), *S. aureus* MTCC 737 (MIC 0.25 mg/ml), *Candida tropicalis* Berkhout, 1923 (Saccharomycetaceae) MTCC 227 (MIC 0.125 mg/ml), and *Candida albicans* Berkhout, 1923 (Saccharomycetaceae) MTCC 227 (MIC 0.25 mg/ml) (Table 2).

#### 3.4.3. Insecticidal Activity

Popularly, in Kenya, the aerial parts of *M. suaveolens* are burned to repel mosquitoes of the species *Anopheles gambiae* [84, 94]. Subsequently, other researchers have highlighted that the oil has biological action against various insects, such as *A. gambiae* itself (Table 3).

Works evaluating the insecticidal action showed that the essential oil of *M. suaveolens* by fumigation showed the effect against *Callosobruchus maculatus* (Fabricius 1775; Coleoptera: Chrysomelidae) (CL_50_ 4.7 *μ*g/ml), *Rhyzopertha dominica* (Fabricius 1972; Coleoptera: Bostrichidae) (CL_50_ 12 *μ*g/ml), *Sitophilus oryzae* (Linnaeus 1763; Coleoptera: Curculionidae) (CL_50_ 10.6 *μ*g/ml), *Tribolium castaneum* (Herbst 1932; Coleoptera: Tenebrionidae) (CL_50_ 23.2 *μ*g/ml) (Tripathi; Upadhyay 2009), and *Sitophilus granarius* (Linnaeus, 1758) (CL_50_ 0.251 *μ*l/insect) [138].

*Mesosphaerum suaveolens* oil showed toxicity through contact and ingestion against Mediterranean flies (*Ceratitis capitata* (Wiedemann, 1824) (Diptera: Tephritidae)) with CL_50_ 13.041 *μ*l/l through ingestion and CL_50_ 0.066 *μ*l/l through contact [30]. Canale et al. [139], when evaluating the toxicity of the oil against *Bactrocera oleae* (Rossi, 1790) (Diptera: Tephritidae), demonstrated that, by ingestion, the product showed a CL_50_ of 4.9 mg/ml. Bezerra et al. [10] evaluated by fumigation the action of the oil and highlighted that it presents great insecticidal action with CL_50_ of 15.5 *μ*g/ml against adults of *Drosophila melanogaster* (Meigen, 1830) (Diptera: Drosophilidae). Also, via the fumigation test, Wangrawa et al. [35] found a CL_50_ of 1.86 *μ*g/ml (oil/air) against *A. gambiae*. Recently, Adjou et al. (2019) found the insecticidal action of *M. suaveolens* oil against *Tenebroides mauritanicus* (Linnaeus, 1758) (Coleoptera: Trogossitidae) with a CL_50_ 0.35 *μ*l/g.

#### 3.4.4. Repellent Activity

Besides causing mortality in insects, the products of *M. suaveolens*, especially the essential oil of the leaves, can repel insects and arachnids of public health and economic interests (Table 4). As for Diptera, the essential oil by fumigation and the fresh leaves by contact were able to repel mosquitoes of the species *Anopheles gambiae*, malaria vector, with the former showing an effective rate of 98% repellency at a low concentration (6%) [152], while the leaves when rubbed on the body had a rate of 66.5% [153]. Besides this Diptera, *Aedes aegypti* vector of arboviruses, such as dengue, yellow fever, chikungunya, and zika, was also repelled when in contact with the ethyl acetate extract of the leaves.

As for insects of economic interest, the leaves of *M. suaveolens* showed that the phytochemicals released by the leaves can repel two species of coleoptera, being *Sitophilus granarius* and *Callosobruchus maculatus*. As for arachnid repellency, the essential oil was able to repel *Amblyomma cajennense* and *Ixodes ricinus*.

#### 3.4.5. Larvicidal Activity

Besides microbiological and insecticidal activities, apolar and polar extracts of *M. suaveolens* showed larvicidal action in some studies (Table 5). Its oil, for example, showed activity against *Aedes albopictus* (Skuse, 1894) (Diptera: Culicidae) (CL_50_ 240.3 *μ*g/ml) [159], *Aedes aegypti* (Linnaeus, 1762) (Diptera: Culicidae) (CL_50_ 0.4 *μ*L/ml) [160], *Artemia salina* (Linnaeus, 1758) (Anostraca: Artemiidae) (CL_50_ 49.72 *μ*g/ml) [10], and *Chrysodeixis chalcites* (Esper, 1789) (Lepidoptera: Noctuidae) (CL_50_ 2.42 *μ*g/ml) [32].

#### 3.4.6. Cytotoxic Activity

Besides microbiological and insecticidal activities, it was evidenced that *M. suaveolens* products present biologically active compounds against cancer cells (Table 6). The activities evaluated using the MTT (3-(4,5-dimethylthiazol-2-yl)-2,5-diphenyltetrazolium bromide) assay demonstrated that the leaves are the source of the anticancer compounds against some cell types. Among these, Lautié et al. [175] evaluated the bioactivities of chlorophore and butanolic extracts against MCF-7 cell lines and showed that such products have low IC_50_ (12 and 2.8 *μ*g/ml, respectively). Besides these cell lines, activities were demonstrated against EAC (Ehrlich ascites carcinoma), human breast epithelial adenocarcinoma (MDA-MB-231), and T-lymphocyte leukemia cells.

Among the studies against cancer cell lines, only the aqueous extract of the leaves showed low cytotoxicity (IC_50_ 1,356.17 *μ*g/ml) when evaluated against T-lymphocyte leukemia-causing cells [176]. Concomitantly in the same study, the ethanolic extract of the same organs had moderate cytotoxicity (IC_50_ 553.52 *μ*g/ml); such fact is explained by the variation of solvent used for the extraction of the compounds.

#### 3.4.7. Other Biological Activities (Antiarachnidic, Antiparasitic, and Molluscicides)

In addition to the aforementioned activities, the herbaceous species present bioactive compounds against other biological organisms. Among these were highlighted parasitic organisms of humans, as reported by Shittu et al. [180], in which evaluating the trypanocidal action (*Trypanosoma brucei brucei*) in vivo of gold nanoparticles from *M. suaveolens* demonstrated that the species can cause a complete clearance of the parasite after seven days of infection. As well as in insecticidal action against malaria vectors (*Anopheles* spp.), *M. suaveolens* exhibits antiplasmodial activity (*Plasmodium falciparum* 3D7) [45, 46, 181]. Noronha et al. [181] evaluated the antiparasitic action of the methanolic extract of *M. suaveolens* leaves and observed that the natural product has an IC_50_ of 3.906 *μ*g/ml against chloroquine-sensitive *Plasmodium falciparum* strains. In the study by Ziegler et al. [45], the researchers isolated a diterpene (dehydroabietinol) from the leaves and observed that the compound was able to inhibit 50% of parasite growth at a low concentration of 7.3 *μ*g/ml. Similarly, Chukwujekwu et al. [46] demonstrated that another diterpene (13*α*-epi-dioxiabiet-8(14)-en-18-ol) isolated from the same organ showed an IC_50_ of 0.11 *μ*g/ml, against *P. falciparum* D10, being of great clinical interest.

Research involving the extracts and essential oil of *M. suaveolens* focus on insecticidal activities, so only one work aimed to evaluate the bioactive potential against Arachnida species [182]. In this study, the authors demonstrated that the essential oil of the leaves at the concentration of 31.3 mg/ml can cause 50% mortality of *Rhipicephalus (Boophilus) microplus* for engorged females, while for juvenile forms of the tick, CL_50_ is 51.6 mg/mL, demonstrating that females are more susceptible to the oil.

Salawu and Odaibo [182] evaluated the molluscicide action of the ethanolic extract of *M. suaveolens* against *Bulinus globosus*, found that the product presents both lethality in adult individuals (LC_50_ 77 *μ*g/ml), and also presents an ovicidal potential (LC_50_ 614 *μ*g/ml).

### 3.5. Pharmacological Activities

#### 3.5.1. Antioxidant Activity

Narayanaswamy and Balakrishnan [84] evaluated the extracts of thirteen plants of ethnomedicinal importance and identified that *M. suaveolens* species among all had the highest activity in both aqueous and ethanolic extracts evaluated. Thus, it demonstrates that the species is a source of compounds of pharmacological interest for the development of natural antioxidant products.

Agarwal [183] showed that the methanolic extract of *M. suaveolens* leaves has potent antioxidant activity evaluated by the 2,2-diphenyl-1-picrylhydrazyl (DPPH) method with IC_50_ value = 40.91 *μ*g/ml, and there is about 69% free radical inhibition capacity at the highest concentration (100 *μ*g/ml). It was also found that the percentage of inhibition is concentration dependent, the higher the concentration the greater the inhibition of free radicals, and that the main phytochemicals involved in this activity may be phenols and flavonoids. The methanolic extract also demonstrated antioxidant activity with assays other than DPPH, such as ferric reducing antioxidant power (FRAP) and 2,2′-azino-bis (3-ethylbenzothiazoline-6-sulfonic acid) (ABTS) [184].

Other studies have also evaluated the free radical scavenging potential by the DPPH method. Gavani and Paarakh [185] evaluated the methanolic extract of *M. suaveolens* leaves and obtained an IC_50_ of 14.04 *μ*g/ml. The aqueous and ethanolic extracts of the fresh leaves of *M. suaveolens* demonstrated high antioxidant activity. The former product had an IC_50_ = 20.32 *μ*g/ml, while the ethanolic extract had a statistically equal result to the positive control, ascorbic acid, with IC_50_ of 7.06 ± 0.82 and 6.69 ± 0.85 *μ*g/mL, respectively [186]. Such results are attributed to the phenolic compounds present in such extracts.

Iqbal et al. [187] reported that the methanolic extract of the seeds of *M. suaveolens* showed better antioxidant activity by the DPPH method (IC_50_ = 72 ± 0.45 *μ*g/ml) when compared to the methanolic extracts of the stem (IC_50_ = 250 ± 5.46 *μ*g/ml) and root (IC_50_ = 143 ± 2.15 *μ*g/ml).

In the work of Priyadharshini and Sujatha [188], four types of leaf extracts were evaluated; in the DPPH assay, three of the extracts had significant results, the best of them being the ethyl acetate extract with a percentage of inhibition (IC_50_ = 137 *μ*g) very close to that of the standard used, ascorbic acid (IC_50_ = 127 *μ*g). In this same study, the acetate extract also stood out with other tests; in the superoxide anion radical scavenging assay, the IC_50_ value = 22.94 *μ*g, and the standard had a value of 21.47 *μ*g.

In addition to leaves, antioxidant potentials of other organs such as flowers were investigated in the study by Banerjee and De [189]. However, the results were not promising, as the extract of the reproductive parts exhibited an IC_50_ of 1690.21 *μ*g/ml against DPPH. Antioxidant studies involving *M. suaveolens* are not restricted to extracts only. Nantitanon et al. [126] evaluated the effect of the essential oil from the leaves; however, it showed low potential to reduce free radicals (IC_50_ of 3.7200 mg/ml). Hsu et al. [190] demonstrated that the seeds also exhibit antioxidant action, as they showed moderate inhibitory activity against xanthine oxidase. In a study conducted by Lima et al. [191], it was observed that the essential oil from *M. suaveolens* leaves showed moderate Fe^2+^ chelating activity at the concentration of 480 *μ*g/ml.

#### 3.5.2. Healing Potential

The healing potential of *M. suaveolens* leaves was evaluated by three types of extracts (petroleum ether, ethanolic, and aqueous) in different wound models (excision, incision, and healing) using albino Wistar rats [192]. In this study, it was revealed that the extract using petroleum ether had the greatest significant effect on wound healing in murine wounds. Shirwaikar et al. [193] evaluated the ethanolic extract on these same three types of wounds, also obtaining significant results which were justified by the free radical scavenging action of this species.

#### 3.5.3. Neuroprotective Activity

Ghaffari et al. [184] determined the neuroprotective potential of the methanolic extract of the aerial parts of *M. suaveolens* on mouse N2A neuroblastoma cells, in which they observed that the natural product inhibits hydrogen peroxide (H_2_O_2_)-induced neuronal death. The authors justified this effect by the fact that the extract can regulate the activation of antioxidant and protective genes of the nerve cells. These results are promising, and the methanolic extract can be employed to treat stress-induced neurodegeneration.

#### 3.5.4. Anti-Inflammatory Activity

Shenoy and Shirwaikar [194] evaluated the anti-inflammatory potential of ethanolic extract of the leaves against inflammation induced by carrageenan in albino rats; the extract showed significant results when compared to standard ibuprofen; this result is justified by the good antioxidant activity of this extract. In the study by Grassi et al. [42], two diterpenes (C_20_) isolated from *M. suaveolens*, suaveolol and methyl suaveolate, showed anti-inflammatory potential when evaluated regarding the reduction of ear edema in rats. In such research, it was observed that the compounds reduced inflammation with ID_50_ = 0.71 *μ*mol/cm^2^ (dose giving 50% edema inhibition) for suaveolol and ID_50_ = 0.60 *μ*mol/cm^2^; however, despite the pharmacological effect, the results were only two to three times less active than the standard drug used in the study indomethacin (ID_50_ = 0.26 *μ*mol/cm^2^).

#### 3.5.5. Antiulcer Activity

*Mesosphaerum suaveolens* leaves are popularly used for the treatment of gastric ulcers; however, no active ingredient had been identified. The first study evaluating such an effect was by Vera-Arvaze et al. [43], in which such authors isolated the diterpene suaveolol from the leaves and evaluated it against an induced experimental model; the results presented indicated that this compound had a gastroprotective effect of more than 70%.

One year after the publication of the mentioned study, Jesus et al. [195] using the ethnopharmacological approach of *M. suaveolens* evaluated its antiulcer potential through the ethanolic extract and its fractions. The results for all products had high significance, *p* > 0.001, with the hexanic fraction of the extract being the most effective with 74% inhibition of induced gastric ulcer at a dose of 500 mg/kg.

#### 3.5.6. Antidiarrheal Activity

Although *M. suaveolens* is popularly used to treat gastrointestinal disorders such as diarrhea, only the work of Shaikat et al. [196] evaluated its antidiarrheal potential. The researchers prepared ethanolic extracts of the leaves and based on the popular use, evaluated against an experimental model of diarrhea induced in mice; the results obtained show that this species presents compounds with the antidiarrheal effect (*p* > 0.001) that may be acting in an isolated or synergistic way.

#### 3.5.7. Antihyperglycemic Activity

The ethanolic extract of *M. suaveolens* leaves was evaluated in the in vivo experimental model of streptozotocin-induced diabetes. The extract at doses of 250 and 500 mg/kg bodyweight was administered orally over 21 days; at the end of the treatment, a decrease in the levels of triglycerides, total cholesterol, and low-density lipoprotein can be seen; these results indicate that this species has significant antidiabetic activity [197].

#### 3.5.8. Hepatoprotective Effect

Ghaffari et al. [198] induced damage to livers of Wistar rats using carbon tetrachloride (CCl4) and subsequently administered doses of 50 and 100 ml/kg of methanolic extract of the aerial parts of *M. suaveolens*. The results were promising, demonstrating that the extract has a hepatoprotective effect, which can be explained by the antioxidant potential of the product, also demonstrated in the study.

#### 3.5.9. Toxicity

A topical cream based on *M. suaveolens* essential oil was prepared based on its popular use in Thailand, where they pointed out further investigations related to its toxicity in humans. This study was conducted on Wistar rats for 28 days under 3, 10, and 30% concentration of essential oil. The results showed us that concentrations of 3 and 10% did not cause a statistically significant dermal toxicity unlike the 30% concentration in which some female rats presented signs of erythema on its shaved dorsal skin between 11 and 14 days after the cream application [199].

## 4. Conclusions

It is evident that *M. suaveolens* is the most studied species of the genus, traditionally used in the new and old world to combat several diseases. Chemically, the species is much investigated, mainly about the composition of its essential oils that can vary according to the locality of occurrence. *Mesosphaerum suaveolens* also behaves as ruderal, and its success may be due to the release of allelochemicals. The species also presents a biotechnological potential corroborated by the remarkable activity against pathogenic microorganisms, insects, and other arthropods that transmit diseases. Finally, the pharmacological applications of the species are highlighted, especially the antioxidant action found in several organs of the species.

## Figures and Tables

**Table 1:** Identified constituents in *Mesosphaerum suaveolens* (L.) Kuntze (Lamiaceae).

Composite	Structure	Identification method	Part of the plant	Citation
Monoterpene				
Sabinene	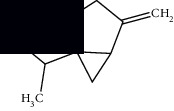	GC-MS	Leaves (essential oil)	[30–35]
Eucalyptol	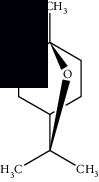	GC-MS	Leaves (essential oil)	[35–37]
Sesquiterpene				
*E*-Caryophyllene	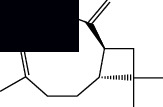	GC-MS	Leaves (essential oil)	[38]
Germacrene D	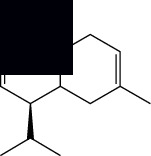	GC-MS	Leaves (essential oil)	[38]
*β*-Caryophyllene	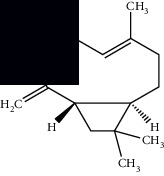	GC-MS	Leaves (essential oil)	[32, 33, 39, 40]
Diterpenes				
Mellowolic acid	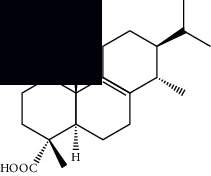	^1^H-NMR and ^13^C-NMR	Leaves and stem	[26, 41]
Suaveolol	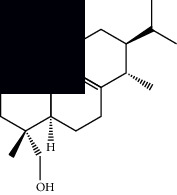	^1^H-NMR and ^13^C-NMR	Leaves	[41–43]
Dehydroabietinol	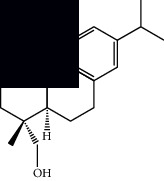	GC-MS, ^1^H-NMR, and ^13^C-NMR	Leaves, stem, and flowers	[29, 44, 45]
9*α*,13*α*-Epi-dioxiabiet-8(14)-en-18-ol	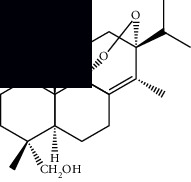	^1^H-NMR and ^13^C-NMR	Leaves	[46]
Methyl suaveolate	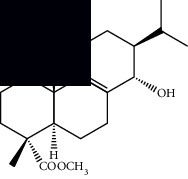	^1^H-NMR and ^13^C-NMR	Leaves	[42]
Ácido 8*α*,9*α*-epoxysuaveolic	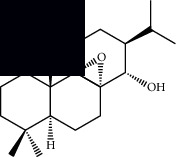	^1^H-NMR and ^13^C-NMR	Leaves and stem	[43]
4*α*-Hydroperoxy-5-enovatodiolide	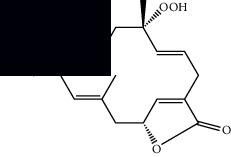	^1^H-NMR and ^13^C-NMR	Flowers	[47]
4-Methylene-5*β*-hydroperoxy ovatodiolide	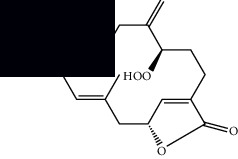	^1^H-NMR and ^13^C-NMR	Flowers	[47]
Ovatodiolide	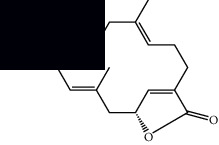	^1^H-NMR and ^13^C-NMR	Flowers	[47]
4*α*-Hydroxy-5-enovatodiolide	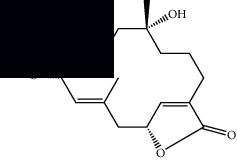	^1^H-NMR and ^13^C-NMR	Flowers	[47]
Phytol	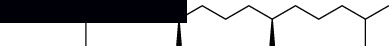	GC-MS	Flowers	[29]
Triterpenes and steroids				
*α*-Peltoboikinolic acid	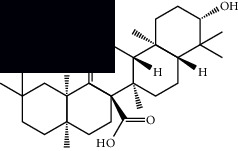	^1^H-NMR and ^13^C-NMR	Root	[48]
Oleanolic acid	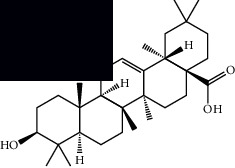	^1^H-NMR and ^13^C-NMR	Root	[48]
Bacosine	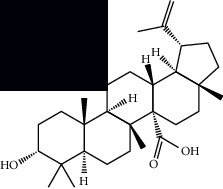	^1^H-NMR and ^13^C-NMR	Root	[49]
Betulinic acid	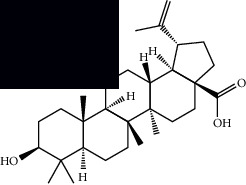	^1^H-NMR and ^13^C-NMR	Root	[49]
Ursolic acid	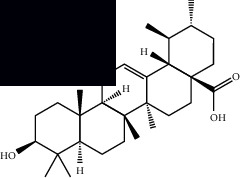	^1^H-NMR and ^13^C-NMR	Root, leaves, and stem	[49, 50]
*α-*Amyrin	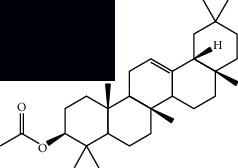	^1^H-NMR and ^13^C-NMR	Root	[51]
3*β*-Hydroxylup-20(29)-en-28-oic acid	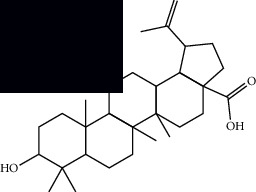	^1^H-NMR and ^13^C-NMR	Root	[51]
Urs-12-en-3*β*-ol-29-oic acid	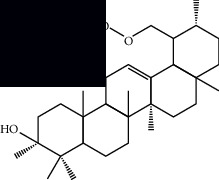	^1^H-NMR and ^13^C-NMR	Leaves and stem	[53]
Lupeol	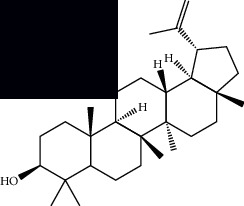	^1^H-NMR and ^13^C-NMR	Leaves	[50]
Rotundic acid	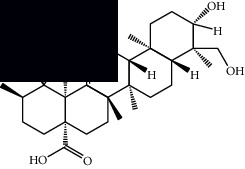	^1^H-NMR and ^13^C-NMR	Leaves and stem	[50]
*β-*Sitosterol	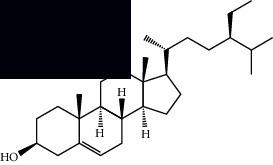	^1^H-NMR and ^13^C-NMR	Leaves and stem	[50]
Hyptadienoic acid	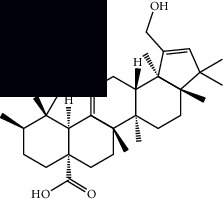	^1^H-NMR and ^13^C-NMR	Leaves and stem	[53]
Phenolics				
Gallic acid	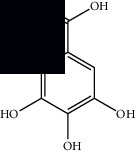	HPLC-DAD and HPTLC	Leaves and stem	[10, 27]
Ellagic acid	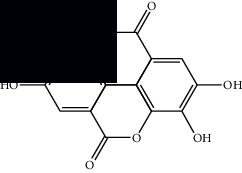	HPLC-DAD	Leaves and aerial parts	[6]
Ferulic acid	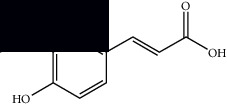	HPTLC	Leaves and stem	[27]
Caffeic acid	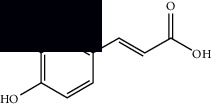	HPLC-DAD and UPLC-MS	Leaves	[6, 10, 54]
Chlorogenic acid	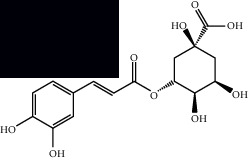	HPLC-DAD and HPTLC	Leaves and stem	[6, 10, 27]
Apigenin	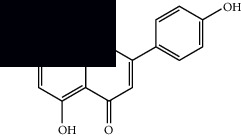	HPLC-DAD	Leaves	[6, 10]
Catechin	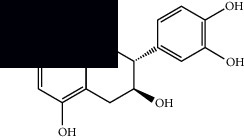	HPLC-DAD	Leaves	[6, 10]
Rutin	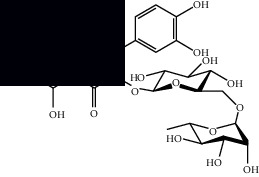	HPLC-DAD and UPLC-MS	Leaves	[6, 10, 54]
Quercetin	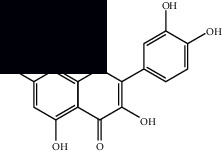	HPLC-DAD and UPLC-MS	Leaves and stem	[6, 10, 27, 54]
Rosmarinic acid	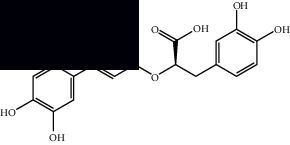	UPLC-MS	Leaves	[54]
Ethyl caffeate	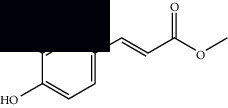	UPLC-MS	Leaves	[54]
Sagerinic acid	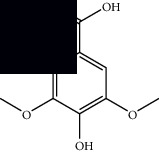	UPLC-MS	Leaves	[54]
Isoquercetin	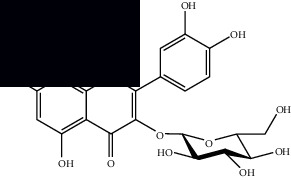	UPLC-MS	Leaves	[54]
Syringic acid	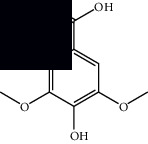	UPLC-MS	Leaves	[54]
Yunnaneic acid	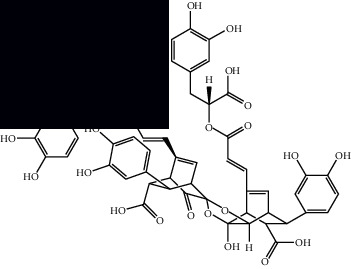	UPLC-MS	Leaves	[54]
Secoiridoid				
Oleoside dimethyl ester	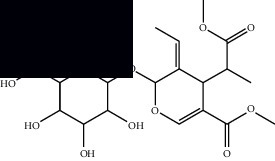	UPLC-MS	Leaves	[54]
Fatty acids				
Linoleic acid	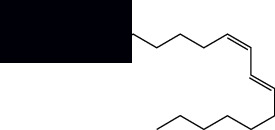	GC-MS	Seeds	[28]
Palmitic acid	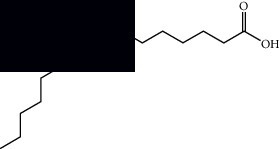	GC-MS	Seeds and leaves	[28, 29]
Oleic acid	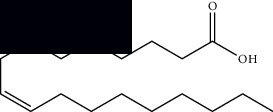	GC-MS	Seeds	[28]
Stearic acid	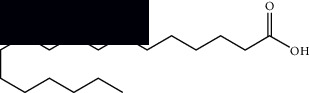	GC-MS	Seeds	[28]
Palmitoleic acid	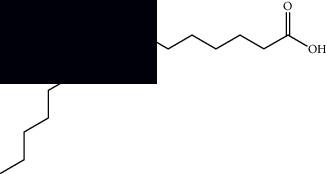	GC-MS	Seeds	[28]
Undecanoic acid	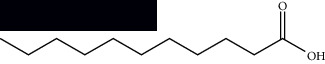	GC-MS	Leaves	[29]
Octanoic acid	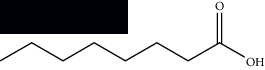	GC-MS	Leaves	[29]
*n*-Decanoic acid	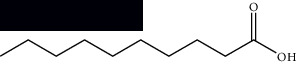	GC-MS	Leaves	[29]
Tetradecanoic acid	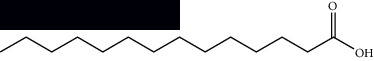	GC-MS	Leaves	[29]
Myristic acid ethyl ester	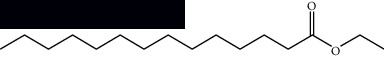	GC-MS	Aerial parts	[55]

**Table 2:** Antimicrobial activities of *Mesosphaerum suaveolens* (L.) Kuntze (Lamiaceae).

Extract/parts of the plant	Assay	Antimicrobial activity	Citation
Methanolic extract of the aerial parts	Disk diffusion test	*Candida albicans* (7 mm)	[105]
Essential oil from the leaves	Disk diffusion test	*Staphylococcus aureus* ATCC 29213 (27 mm) *Escherichia coli* ATCC 25922 (22 mm) *Trichophyton mentagrophytes* (15 mm) *Trichophyton rubrum* (24 mm)	[106]
Essential oil from the leaves	Dilution method	*Staphylococcus aureus* ATCC 29213 (MIC 8.82 mg/ml) *Streptococcus pyogenes* P183 (MIC 4.41 mg/ml) *Streptococcus pyogenes* P31 P183 (MIC 4.41 mg/ml)	[106]
Essential oil from the leaves	Disk diffusion test	*Bacillus subtilis* (MIC 16.67 *μ*l/ml) *Staphylococcus aureus* (MIC 1.56 *μ*l/ml) *Escherichia coli* (MIC 6.25 *μ*l/ml) *Proteus vulgaris* (MIC 50 *μ*l/ml) *Pseudomonas aeruginosa* (MIC 25 *μ*l/ml)	[107]
Essential oil from the leaves	Disk diffusion test and dilution method	*Erwinia herbicola* MTCC 3609 (MIC 2 *μ*l/ml) *Pseudomonas putida* MTCC 1190 (MIC 8 *μ*l/ml)	[108]
Essential oil from the aerial parts	Microdilution method	*Staphylococcus aureus* CMCC 26001 (MIC 50 *μ*g/ml) *Bacillus subtilis* CMCC 63501 (MIC 25 *μ*g/ml) *Pseudomonas aeruginosa* CMCC 10104 (MIC 50 *μ*g/ml) *Escherichia coli* CMCC 44102 (MIC 25 *μ*g/ml) *Fusarium graminearum* (MIC 50 *μ*g/ml) *Botrytis cinerea* (MIC 25 *μ*g/ml) *Exerohilum turcicum* (MIC 50 *μ*g/ml) *Lecanosticta acicola* (MIC 50 *μ*g/ml)	[104]
Ethanolic extract of the aerial parts	Disk diffusion test	*Aeromonas caviae* (0.1 mm) *Aeromonas hydrophila* (0.1 mm) *Ralstonia* spp. (0.17 mm) *Shigella* spp. (0.23 mm)	[109]
Aerial parts essential oil	Disk diffusion test	*Trichophyton mentagrophyte* (15.7 mm) *Trichophyton rubrum* (14.7 mm) *Microsporum gypeum* (13.3 mm) *Candida albicans* (13.7 mm)	[110]
Blossoms essential oil	Disk diffusion test	*Escherichia coli* ATCC 25922 (MIC 350 *μ*l/ml) *Klebsiella pneumoniae* ATCC 23357 (MIC 400 *μ*l/ml) *Salmonella typhi* CDC57 (MIC 450 *μ*l/ml)	[111]
Flower essential oil	Disk diffusion test	*Escherichia coli* ATCC 25922 (MIC 350 *μ*l/ml) *Klebsiella pneumoniae* ATCC 23357 (MIC 400 *μ*l/ml) *Salmonella typhi* CDC57 (MIC 450 *μ*l/ml)	[111]
Leaf hydroethanolic extract (70%)	Agar diffusion method	*Staphylococcus aureus* Meti-R (MIC 3.12 mg/ml) *Pseudomonas aeruginosa* Cefta/Imp-R (MIC 12.5 mg/ml) *Staphylococcus aureus* ATCC 25923 (MIC 3.12 mg/ml) *Pseudomonas aeruginosa* ATCC 27853 (MIC 3.12 mg/ml)	[103]
Leaves essential oil	Agar diffusion method	*Staphylococcus aureus* Meti-R (MIC 5.37 mg/ml) *Pseudomonas aeruginosa* Cefta/Imp-R (MIC 10.75 mg/ml) *Staphylococcus aureus* ATCC 25923 (MIC 5.37 mg/ml) *Pseudomonas aeruginosa* ATCC 27853 (MIC 10.75 mg/ml)	[103]
Seed fixed oil	Dilution method	*Escherichia coli* MTCC 443 (MIC 0.5 mg/ml) *Salmonella typhi* MTCC 531 (MIC 0.125 mg/ml) *Shigella flexneri* MTCC 1457 (MIC 0.5 mg/ml) *Vibrio vulnificus* MTCC 1145 (MIC 0.5 mg/ml) *Pseudomonas aeruginosa* MTCC 424 (MIC 0.125 mg/ml) *Lactobacillus plantarum* MTCC 2621 (MIC 0.125 mg/ml) *Lactobacillus leishmanii* MTCC 911 (MIC 0.25 mg/ml) *Staphylococcus aureus* MTCC 737 (MIC 0.25 mg/ml) *Candida tropicalis* MTCC 227 (MIC 0.125 mg/ml) *Candida albicans* MTCC 227 (MTCC 0.25 mg/ml)	[112]
Essential oil from leaves	Disc diffusion method	*Escherichia coli* ATCC 25922 (MIC 350 *μ*l/ml) *Klebsiella pneumoniae* ATCC 23357 (MIC 300 *μ*l/ml) *Salmonella typhi* CDC57 (MIC 400 *μ*l/ml)	[111]
Hexanic extract from the seeds	Microdilution method	*Staphylococcus aureus* Meti-R (MIC 0.375 mg/ml) *Enterococcus faecalis* ATCC 29212 (MIC 0.1875 mg/ml) *Escherichia coli* ATCC 25922 (MIC 0.1875 mg/ml) *Pseudomonas aeruginosa* ATCC 15442 (MIC 0.375 mg/ml) *Klebsiella pneumoniae* (MIC 0.375 mg/ml) *Acinetobacter baumannii* (MIC 0.375 mg/ml)	[113]
Aqueous extract from the leaves	Microdilution method	*Candida albicans* 77 (IC_50_ 266.4 *μ*g/ml) *Candida albicans* 40006 (IC_50_ 300.4 *μ*g/ml) *Candida tropicalis* 23 (IC_50_ 359.9 *μ*g/ml) *Candida tropicalis* 40042 (IC_50_ 640.3 *μ*g/ml)	[6]
Aqueous extract from aerial parts	Microdilution method	*Candida albicans* 77 (IC_50_ 18.5 *μ*g/ml) *Candida albicans* 40006 (IC_50_ 526.4 *μ*g/ml) *Candida tropicalis* 23 (IC_50_ 25 *μ*g/ml) *Candida tropicalis* 40042 (IC_50_ 58.62 *μ*g/ml)	[6]
Essential oil from the leaves	Disk diffusion test	*Staphylococcus aureus* UCH 511 (14 mm) *Bacillus cereus* (10 mm) *Escherichia coli* NCTC 7001 (12 mm) *Pseudomonas aeruginosa* UCH 655 (14 mm) *Candida albicans* (16 mm)	[114]
Leaf ethanolic extract	Disk diffusion test	*Sclerotium rolfsii* (MIC 2000 mg/ml)	[115]
Leaf hydroethanolic extract (70%)	Disk diffusion test	*Staphylococcus aureus* Meti-R (24 mm) *Staphylococcus aureus* ATCC 25923 (24 mm) *Pseudomonas aeruginosa* Ceft/Imp-R (16 mm) *Pseudomonas aeruginosa* ATCC 27853 (20 mm)	[116]
Leaf essential oil	Disk diffusion test	*Staphylococcus aureus* Meti-R (13 mm) *Staphylococcus aureus* ATCC 25923 (16 mm) *Pseudomonas aeruginosa* Ceft/Imp-R (0 mm) *Pseudomonas aeruginosa* ATCC 27853 (0 mm)	[116]
Leaf essential oil	Disk diffusion test	*Staphylococcus aureus* (MIC 30 *μ*g/ml) *Bacillus subtilis* (MIC 26 *μ*g/ml) *Proteus vulgaris* (MIC 15 *μ*g/ml) *Candida albicans* (MIC 10 *μ*g/ml) *Aspergillus niger* (MIC 40 *μ*g/ml) *Pseudomonas aeruginosa* (MIC 28 *μ*g/ml) *Trichophyton mentagrophytes* (MIC > 100 *μ*g/ml) *Escherichia coli* (MIC 26 *μ*g/ml) *Klebsiella pneumoniae* (MIC 37 *μ*g/ml) *Yersinia enterocolitica* (MIC > 100 *μ*g/ml)	[117]
Essential oil from the leaves	Disk diffusion test	*Aspergillus flavus* (MIC 93.8 *μ*l/ml)	[118]
Essential oil from the leaves	Disk diffusion test	*Escherichia coli* (7 mm) *Bacillus subtilis* (8 mm) *Vibrio cholerae* (9 mm) *Shigella dysenteriae* (9 mm) *Corynebacterium diphtheriae* (10 mm) *Salmonella typhi* (9 mm) *Streptococcus faecalis* (9 mm) *Bacillus pumilus* (9 mm) *Streptococcus pyogenes* (8 mm) *Micrococcus* (8 mm) *Pseudomonas solanacearum* (10 mm)	[119]
Crushed leaves	Disk diffusion test	*Aspergillus flavus* (56% inhibition at 5% concentration)	[120]
Methanolic extract from the leaves	Disk diffusion test	*Bacillus cereus* (9 mm) *Bacillus subtilis* (3 mm)*Bacillus megaterium* (1 mm)*Staphylococcus aureus* (6 mm) *Enterococcus faecalis* (7 mm)*Escherichia coli* (12 mm) *Salmonella typhi* (6 mm)*Salmonella paratyphi* (5 mm)*Pseudomonas aeruginosa* (3 mm)*Klebsiella pneumoniae* (4 mm)	[121]
Essential oil from the leaves	Microdilution method	*Saccharomyces cerevisiae* (IC_50_ 1000 *μ*g/ml) *Fusarium moniliforme* 7075 (IC_50_ > 1500 *μ*g/ml) *Mucor* sp. (IC_50_ 750 *μ*g/ml)	[122]
Whole plant aqueous extract	Disk diffusion test	*Candida albicans* (0 mm) *Colletotrichum capsici* (10 mm) *Fusarium oxysporum* F.sp. *lycopersici* (5 mm) *Klebsiella pneumoniae* (14 mm) *Escherichia coli* (12 mm) *Staphylococcus aureus* (0 mm) *Pseudomonas aeruginosa* (0 mm)	[123]
Whole plant ethanolic extract	Disk diffusion test	*Candida albicans* (19 mm) *Colletotrichum capsici* (20 mm) *Fusarium oxysporum* F.sp. *lycopersici* (18 mm) *Klebsiella pneumoniae* (22 mm) *Escherichia coli* (18 mm) *Staphylococcus aureus* (29 mm) *Pseudomonas aeruginosa* (12 mm)	[123]
Ethanolic (1), chloroform (2), methanolic (3), petroleum ether (4), and aqueous (5) extracts of the whole plant	Disk diffusion test	*Aspergillus niger*: 1: 6 mm 2: 11 mm 3: 0.5 mm 4: 7 mm 5: 12 mm *Candida albicans*: 1: 10 mm 2: 10.5 mm 3: 3.5 mm 4: 8.5 mm 5: 8 mm *Fusarium* sp.: 1: 12 mm 2: 11 mm 3: 3 mm 4: 12 mm 5: 19 mm	[124]
Essential oil from the leaves	Microdilution method	*Aspergillus fumigatus* ATCC 40640 (MIC 40 *μ*l/ml) *Aspergillus parasiticus* ATCC 15517 (MIC 40 *μ*l/ml)	[36]
Essential oil from the leaves	Microdilution method	*Trichophyton mentagrophytes* (MIC 640 *μ*l/ml) *Staphylococcus aureus* (MIC 160 *μ*l/ml) *Streptococcus suis* (MIC 160 *μ*l/ml) *Erysipelothrix rhusiopathiae* (MIC 80 *μ*l/ml) *Actinomyces pyogenes* (MIC 80 *μ*l/ml) *Pasteurella multocida* (MIC 80 *μ*l/ml) *Pseudomonas aeruginosa* (MIC 20 *μ*l/ml) *Escherichia coli* (MIC 20 *μ*l/ml)	[125]
Essential oil from the leaves	Disk diffusion test	*Rhizoctonia solani* (MIC 4000 *μ*l/l) *Pythium debaryanum* (MIC 300 *μ*l/l) *Pythium aphanidermatum* (MIC 300 *μ*l/l)	[126]
Aqueous extract of the leaves	Microdilution method	*Trichoderma viride* (IC_50_ > 500 *μ*l/l) *Alternaria porri* (IC_50_ > 500 *μ*l/l) *Aspergillus parasiticus* (IC_50_ > 500 *μ*l/l) *Aspergillus fumigatus* (IC_50_ > 500 *μ*l/l) *Fusarium lini* (IC_50_ > 500 *μ*l/l) *Alternaria brassicae* (IC_50_ > 500 *μ*l/l) *Colletotrichum* sp. (IC_50_ > 500 *μ*l/l) *Fusarium sesami* (IC_50_ > 500 *μ*l/l) *Fusarium nivale* (IC_50_ > 500 *μ*l/l) *Alternoria solani* (IC_50_ > 500 *μ*l/l) *Fusarium oxysporum* f. sp. *ciceri* (IC_50_ > 393.4 *μ*l/l) *Aspergillus niger* (IC_50_ > 244.7 *μ*l/l) *Aspergillus sulphureus* (IC_50_ > 475.4 *μ*l/l) *Helminthosporium oryzae* (IC_50_ > 197.7 *μ*l/l) *Alternoria alternata* (IC_50_ > 475.5 *μ*l/l) *Alternoria tenuissima* (IC_50_ > 321.1 *μ*l/l) *Fusarium semitectum* (IC_50_ > 243.5 *μ*l/l) *Cladosporium cladosporioides* (IC_50_ > 228.6 *μ*l/l) *Drechslera aunti* (IC_50_ > 411 *μ*l/l) *Penicillium citrinum* (IC_50_ > 446.7 *μ*l/l)	[127]
Methanolic extract of the leaves	Disk diffusion test	*Staphylococcus aureus* (6 mm) *Bacillus subtilis* (9 mm)	[128]
Hexanic extract of the leaves	Microdilution method	*Escherichia coli* ATCC 10536 (IC_50_ ≅ 200 *μ*g/ml) *Proteus mirabilis* ATCC 7002 (IC_50_ ≅ 200 *μ*g/ml) *Klebsiella pneumoniae* ATCC 10031 (IC_50_ ≅ 400 *μ*g/ml) *Proteus vulgaris* ATCC 49132 (IC_50_ ≅ 100 *μ*g/ml)*Serratia marcescens* FJ584421 (IC_50_ ≅ 50 *μ*g/ml)	[29]
Petroleum ether (1), chloroform (2), methanolic (3), and aqueous (4) extracts of the leaves	Disk diffusion test	*Staphylococcus aureus* ATCC 29213: 1: 13 mm 2: 8 mm 3: 8 mm 4: 12 mm *Listeria monocytogenes* ATCC 19115: 1: 0 mm 2: 7 mm 3: 5 mm 4: 0 mm *Escherichia coli* ATCC 25922: 1: 7 mm 2: 10 mm 3: 0 mm 4: 0 mm *Serratia marcescens* ATCC 21074: 1: 18 mm 2: 0 mm 3: 8 mm 4: 0 mm *Aspergillus flavus* ATCC 32612: 1: 18 mm 2: 0 mm 3: 0 mm 4: 14 mm	[129]
Leaf essential oil	Disk diffusion test	*Fusarium oxysporum* f. sp. *gladioli* MPPLU 01 (MIC 0.6 *μ*l/ml)	[130]
Leaf essential oil	Disk diffusion test	*Rhizoctonia solani* (MIC 5.000 *μ*g/ml) *Sclerotinia sclerotiorum* (MIC 5.000 *μ*g/ml) *Sclerotium rolfsii* (MIC 5.000 *μ*g/ml)	[131]
Leaf methanolic extract	Disk diffusion test	*Klebsiella pneumoniae* MTCC 109 (7.5 mm)*Bacillus subtilis* MTCC 121 (8 mm)*Salmonella typhi* MTCC 733 (7 mm)	[132]
Leaf essential oil	Disk diffusion test	*Fusarium oxysporum* f.sp. *gladioli* MPPLU 01 (MIC 0.998 *μ*g/ml)	[133]
Aerial parts methanolic extract	Disk diffusion test	*Candida albicans* (7 mm)	[105]
Leaf essential oil	Disk diffusion test	*Microsporum gypseum* (MIC 625 *μ*g/ml) *Aureobasidium pullulans* (MIC 5.000 *μ*g/ml) *Candida albicans* (MIC 10.000 *μ*g/ml) *Aspergillus flavus* (MIC 10.000 *μ*g/ml)*Trichophyton rubrum* (MIC 156 *μ*g/ml)*Cryptococcus neoformans* (MIC 5.000 *μ*g/ml)	[134]
Essential oil from the leaves	Microdilution method	*Staphylococcus aureus* (MIC 64 and 256 *μ*g/ml) *Candida albicans* (IC_50_ 18.15 *μ*g/ml) *Candida tropicalis* (IC_50_ 40.4 *μ*g/ml)	[135]
Leaf hydroethanolic extract	Disk diffusion test	*Staphylococcus aureus* (0.7 cm) *Salmonella typhi* (0.52 cm) *Escherichia coli* (0.25 cm)	[136]
Leaf essential oil	Microdilution method	*Enterococcus faecalis* (IC_50_ 5.78 *μ*g/ml) *Staphylococcus aureus* (IC_50_ 68.98 *μ*g/ml) *Bacillus cereus* (IC_50_ 9.35 *μ*g/ml) *Candida albicans* (IC_50_ 6.78 *μ*g/ml)	[137]

**Table 3:** Insecticidal activities of *Mesosphaerum suaveolens* (L.) Kuntze (Lamiaceae).

Extract/parts of the plant	Assay	Insecticidal activity	Citation
Leaf essential oil	Fumigation Ingestion Contact toxicity	*Ceratitis capitata* Fumigation: CL_50_ 18.37 *μ*l/l Ingestion: CL_50_ 13.041 *μ*l/l Contact toxicity: CL_50_ 0.066 *μ*l/l	[30]
Leaf essential oil	Fumigation	*Callosobruchus maculatus* (CL_50_ 4.7 *μ*g/ml) *Rhyzopertha dominica* (CL_50_ 12 *μ*g/ml) *Sitophilus oryzae* (CL_50_ 10.6 *μ*g/ml) *Tribolium castaneum* (CL_50_ 23.2 *μ*g/ml) *Callosobruchus maculatus* (CL_50_ 57 *μ*g/mg) *Rhyzopertha dominica* (CL_50_ 126 *μ*g/mg) *Sitophilus oryzae* (CL_50_ 101 *μ*g/mg) *Tribolium castaneum* (CL_50_ 167 *μ*g/mg)	[34]
Leaf essential oil	Contact toxicity	*Sitophilus granarius* (CL_50_ 0.251 *μ*L/insect)	[138]
Leaf essential oil	Ingestion	*Bactrocera oleae* (CL_50_ 4.9 mg/ml)	[139]
Leaf methanolic extract	Contact	*Sitophilus oryzae* (CL_50_ 475.9 *μ*l/ml) *Sitophilus zeamais* CL_50_ 707.4 *μ*l/ml) *Callosobruchus maculatus* (CL_50_ 451.2 *μ*l/ml)	[140]
Leaf essential oil	Fumigation	*Tribolium castaneum* (CL_50_ 229.33 *μ*g/ml)	[141]
Leaf essential oil	Fumigation	*Drosophila melanogaster* (CL_50_ 15.5 *μ*g/ml)	[10]
Leaf infusion	Ingestion	*Drosophila melanogaster* (CL_50_ > 30.3 *μ*g/ml)	[10]
Leaf essential oil	Fumigation	*Anopheles gambiae* (CL_50_ 1.86 *μ*g/ml)	[35]
Leaf essential oil	Contact toxicity	*Tenebroides mauritanicus* (CL_50_ 0.35 *μ*l/g)	[142]
Leaf essential oil	Toxicity by ingestion	*Apis mellifera* (CL_50_ 43.03%)	[143]
Leaf ethanolic extract	Fumigation	*Callosobruchus maculatus* (100% mortality (at 10% concentration)	[144]
Crushed leaves	Ingestion toxicity	*Callosobruchus maculatus* (CL_50_ 73 mg/g)	[145]
Crushed leaves and flowers	Ingestion toxicity	*Callosobruchus maculatus* (20.1% of mortality)	[146]
Crushed leaves	Ingestion toxicity	*Callosobruchus maculatus* (96.6% of mortality in the concentration 43.75 mg/g) *Sitophilus zeamais* (30% of mortality in the concentration 43.75 mg/g)	[147]
Leaf essential oil	Fumigation	*Callosobruchus maculatus* (CL_50_: 1.3 *μ*l/l)	[148]
Leaf essential oil	Fumigation	*Callosobruchus maculatus* (CL_50_: >20 *μ*l/l)	[149]
Leaf essential oil	Fumigation	*Callosobruchus maculatus* (CL_50_: >2.6 *μ*l/ml)	[150]
Leaf essential oil	Fumigation	*Nauphoeta cinerea* (did not show insecticidal action)	[151]

**Table 4:** Repellent activities of *Mesosphaerum suaveolens* (L.) Kuntze (Lamiaceae).

Extract/parts of the plant	Assay	Insecticidal activity	Citation
Aerial parts essential oil	Contact	*Amblyomma cajennense* (EC_50_ 0.55 mg/cm²)	[23]
Crushed leaves	Fumigation	*Callosobruchus maculatus* (repellency from 5 minutes of exposure)	[146]
Leaf essential oil	Fumigation	*Anopheles gambiae* (98% repellency rate at 6% concentration)	[152]
Fresh leaves	Contact	*Anopheles gambiae* (repellency rate 66.5%)	[153]
Leaf essential oil	Fumigation	*Sitophilus granarius* (repellency rate 40% at 100% concentration)	[154]
Leaf ethyl acetate extract	Contact	*Aedes aegypti* (repellency rate of 78.8% for 500 *μ*l)	[155]
Crushed leaves (1) and twigs (2)	Contact	*Callosobruchus maculatus*: 1: repellency index: 0.11 2: repellency index: 0.2	[156]
Leaf essential oil	Fumigation	*Anopheles* sp. (67% repellency rate at 6.3 *μ*g/ml concentration)	[157]
Leaf essential oil	Fumigation	*Ixodes ricinus* (repellency rate 93% at 10% concentration)	[158]

**Table 5:** Larvicidal activities of *Mesosphaerum suaveolens* (L.) Kuntze (Lamiaceae).

Extract/parts of the plant	Assay	Larvicidal activity	Citation
Leaf essential oil	Acute toxicity	*Artemia salina* (CL_50_ 49.72 *μ*g/ml)	[10]
Leaf infusion	Acute toxicity	*Artemia salina* (CL_50_ > 1000 *μ*g/ml)	[10]
Leaf essential oil	Acute toxicity	*Chrysodeixis chalcites* (CL_50_ 2.42 *μ*g/ml)	[32]
Essential oil from leaves	Acute toxicity	*Aedes albopictus* (CL_50_ 240.3 *μ*g/ml)	[159]
Leaf essential oil	Acute toxicity	*Aedes aegypti* (CL_50_ 0.4 *μ*L/ml)	[160]
Hexanic extract (1), diethyl ether (2), dichloromethane (3), and ethyl acetate (4) from leaves	Acute toxicity	*Aedes aegypti* 1: CL_50_ 543.66 *μ*g/ml 2: CL_50_ 1443.53 *μ*g/ml 3: CL_50_ 1292.36 *μ*g/ml 4: CL_50_ 853.04 *μ*g/ml	[161]
Hexanic extract (1), diethyl ether (2), dichloromethane (3), and ethyl acetate (4) from aerial parts	Acute toxicity	*Anopheles stephensi* 1: CL_50_ 1.52 mg/ml 2: CL_50_ 1.49 mg/ml 3: CL_50_ 1.39 mg/ml 4: CL_50_ 0.94 mg/ml	[162]
Hexanic (1), isopropanolic (2), methanolic (3), acetone (4), and dimethyl sulfoxide (5) extract from the leaves	Acute toxicity	*Aedes albopictus* 1: CL_50_ 1.52 mg/ml 2: CL_50_ 1.52 mg/ml 3: CL_50_ 1.52 mg/ml 4: CL_50_ 1.52 mg/ml 5: CL_50_ 1.52 mg/ml	[163]
Isopropanolic (1), methanolic (2), acetonic (3), dimethyl sulfoxide (4), and aqueous extract from the leaves (5)	Acute toxicity	*Aedes albopictus* 1: CL_50_ 900 *μ*g/ml 2: CL_50_ 940 *μ*g/ml 3: CL_50_ 820 *μ*g/ml 4: CL_50_ 1590 *μ*g/ml 5: CL_50_ 1560 *μ*g/ml	[163]
Aqueous leaf extract	Acute toxicity	*Anopheles stephensi* (CL_50_ 26.08 mg/ml) *Aedes aegypti* (CL_50_ 17.6 mg/ml) *Culex quinquefasciatus* (CL_50_ 33.68 mg/ml)	[164]
Methanolic (1), chloroform (2), and petroleum ether (3) extracts of the leaves	Acute toxicity	*Culex quinquefasciatus*: 1: CL_50_ > 300 *μ*g/ml 2: CL_50_ 41.93 *μ*g/ml 3: CL_50_ 38.39 *μ*g/ml *Aedes aegypti*: 1: CL_50_ > 300 *μ*g/ml 2: CL_50_ > 300 *μ*g/ml 3: CL_50_ 64.49 *μ*g/ml	[165]
Dichloromethane extract from the aerial parts	Acute toxicity	*Anopheles gambiae* (CL_50_ 63.5 mg/ml)	[166]
Aqueous extract from aerial parts	Spraying	*Sesamia calamistis* (37.85% motility (300 mg/ml))	[167]
Aqueous extract from leaves and fruits	Contact toxicity	*Mussidia nigrivenella* (49% motility (200 mg/g)) *Sesamia calamistis* (37% motility (200 mg/g)) *Eldana saccharina* (41% motility (200 mg/g))	[168]
Hexanic (1), chloroform (2), ethyl acetate (3), and methanolic (4) extract from the aerial parts	Acute toxicity	*Culex quinquefasciatus*: 1: CL_50_ 213.09 *μ*g/ml 2: CL_50_ 217.64 *μ*g/ml 3: CL_50_ 167.59 *μ*g/ml 4: CL_50_ 86.93 *μ*g/ml	[169]
Crushed aerial parts	Contact toxicity	*Helicoverpa armigera* (66.7% mortality at 20% concentration)	[170]
Essential oil from leaves	Ingestion toxicity	*Spodoptera frugiperda* (CL_50_ 600 *μ*g/ml)	[171]
Hexanic extract (1), chloroform (2), and ethyl acetate (3) from leaves	Ingestion toxicity	*Helicoverpa armigera*: 1: mortality of 31.68% (concentration: 1%) 2: mortality of 65.28% (concentration: 1%) 3: mortality of 72.87% (concentration: 1%) *Spodoptera litura*: 1: mortality of 34.09% (concentration: 1%) 2: mortality of 63.62% (concentration: 1%) 3: mortality of 74.56% (concentration: 1%) *Earias vittella*: 1: mortality of 44.37% (concentration: 1%) 2: mortality of 69.28% (concentration: 1%) 3: mortality of 73.40% (concentration: 1%) *Leucinodes orbonalis*: 1: mortality of 37.57% (concentration: 1%) 2: mortality of 67.62% (concentration: 1%) 3: mortality of 78.28% (concentration: 1%)	[172]
Essential oil from leaves (1) and crushed leaves (2)	Contact toxicity	*Dinarmus basalis*: 1: CL_50_ 7.76 *μ*l 2: CL_50_ 14.06 g	[173]
Leaf essential oil	Acute toxicity	*Aedes aegypti* (CL_50_ 139.07 *μ*g/ml)	[174]

**Table 6:** Cytotoxic activities of *Mesosphaerum suaveolens* (L.) Kuntze (Lamiaceae).

Extract/parts of the plant	Assay	Cytotoxic activity	Citation
Chloroform (1) and butanolic (2) extract from the leaves	MTT assay	MCF-7 cell lines 1: IC_50_ 12 *μ*g/ml 2: IC_50_ 2.8 *μ*g/ml	[175]
Ethanolic (1) and aqueous (2) extracts of the leaves	MTT assay	Human T-lymphocyte leukemia: 1: IC_50_ 553.52 *μ*g/ml 2: IC_50_ 1356.17 *μ*g/ml	[176]
Ethanolic extract of the aerial parts	MTT assay	EAC cells (IC_50_ 10.63 *μ*g/ml)	[177]
Leaf callus	MTT assay	Human breast epithelial adenocarcinoma (MDA-MB-231) IC_50_ 74.66 *μ*g/ml Prostate cancer (PC-3) IC_50_ 173.21 *μ*g/ml	[178]
Silver nanoparticles from the leaves	MTT assay	Human breast epithelial adenocarcinoma (MDA-MB-231) IC_50_ 63.16 *μ*g/ml Prostate cancer (PC-3) IC_50_ 52.49 *μ*g/ml	[179]

## Data Availability

The data used to support the findings of this study are available from the corresponding author upon request.
